# Rationale for the Successful Management of EDTA Chelation Therapy in Human Burden by Toxic Metals

**DOI:** 10.1155/2016/8274504

**Published:** 2016-11-08

**Authors:** Maria Elena Ferrero

**Affiliations:** Dipartimento di Scienze Biomediche per la Salute, Università degli Studi di Milano, Via Mangiagalli 31, Milan, Italy

## Abstract

Exposure to environmental and occupational toxicants is responsible for adverse effects on human health. Chelation therapy is the only procedure able to remove toxic metals from human organs and tissue, aiming to treat damage related to acute and/or chronic intoxication. The present review focuses on the most recent evidence of the successful use of the chelating agent ethylenediaminetetraacetic acid (EDTA). Assessment of toxic-metal presence in humans, as well as the rationale of EDTA therapy in cardiovascular and neurodegenerative diseases, is reported.

## 1. Introduction

The present review considers the acute and chronic treatment of toxic-metal burden with ethylenediaminetetraacetic acid (EDTA), as well as the clinical importance of other toxic-metal chelators and iron chelators.

### 1.1. Environmental Pollution Effects on Metal Intake

Toxic metals can provoke numerous adverse effects on human health. Individuals may be exposed to toxic metals present in the environment via multiple routes, such as the respiratory tract through inhalation of air pollution [1], or orally by ingestion of contaminated food and water [2]. Environmental exposure represents a health risk for the general population and more specifically for some professional categories.

#### 1.1.1. High Risk Workers

Recently, a relationship between blood metals and inflammation has been seen in taxi drivers [3]. These workers showed increased whole blood concentration of mercury (Hg), arsenic (As), lead (Pb), and cadmium (Cd) compared to controls; serum inflammatory markers, such as interleukin 1*β*, interleukin 6, and tumor necrosis factor (TNF) *α*, showed an increase. Homocysteine levels in these workers were significantly higher [hyperhomocysteinemia is a well-known risk factor for cardiovascular disease (CVD)] [4], while glutathione peroxidase (GPX) activity and renal function were impaired. These results suggest that Hg, As, Pb, and Cd can be considered important contributors to the development of CVD. In particular, the role of Hg toxicity in the pathogenetic mechanisms of hypertension, atherosclerosis, coronary heart disease, myocardial infarction, cardiac arrhythmias, heart-rate variability, sudden death, cerebrovascular accidents, carotid artery disease, renal dysfunction, and total mortality has already been highlighted [5]. A common mechanism of the damage provoked by toxic metals seems to be due to the induction of oxidative stress. Oxidative stress is provoked by imbalanced redox states, involving either excessive generation of reactive oxygen species (ROS) or dysfunction of the antioxidant system. For example, Pb and Cd have a high affinity for –SH groups in enzymes of the antioxidative defense system, such as superoxide dismutase (SOD), catalase (CAT), GPX, and glucose-6-phosphate dehydrogenase (G6PD), and subsequently inhibit their activity. Apart from targeting -SH groups, Pb and Cd, as divalent cations, can also replace divalent bioelements that serve as important cofactors of antioxidant enzymes, such as GPX, SOD, and CAT, resulting in their inactivation. It has also been confirmed that both metals affect levels of glutathione (GSH), a tripeptide that contains more than 90% of the nontissue sulfur in the human body, representing one of the most important components of antioxidant protection. So, both Pb and Cd induce the generation of ROS and depletion of the antioxidant defense system [6].

Another professional category, coke-oven workers, has been examined: in these subjects, the interaction of heavy metals (As, Cd, chromium or Cr, Nickel or Ni, and Pb) and polycyclic aromatic hydrocarbons that increases oxidative stress has been demonstrated [7]. Moreover, human exposure to Pb compounds has been seen to cause liver enlargement and to activate inflammatory reactions characterized by moderate cholestasis within the bile ducts; these conditions are more evident in subjects with higher Pb exposure levels [8].

#### 1.1.2. Toxicity in relation to Geographic Areas

In general, man-made chemicals, including xenoestrogens, pesticides, other than heavy metals, and an unhealthy lifestyle, mainly tobacco smoking, alcohol consumption, and medical-drug abuse, are considered the major factors causing poor prenatal development through the generation of ROS and cellular oxidative damage [9]. The risks related to toxic-metal exposure differ in relation to the geographical location. Indeed, in some areas, toxicological health risks seem to be higher than in others: in Haiti, ground water shows Pb and Cr contamination [10], whereas significant levels of As, Pb, Cd, Ni, and Cr have been found in edible fish tissue in the Pearl River Delta in China [11]. Furthermore, environmental contaminants, for example, toxic metals such as aluminum (Al), are considered a possible cause of Alzheimer's disease [12].

#### 1.1.3. Effects on Pregnancy and Childhood

Heavy metal exposure during pregnancy is potentially harmful to the developing fetus. A recent study focused on prenatal contamination by examining Pb, Hg, and Cd concentrations in blood samples from newborns (a specimen of umbilical-cord blood was drawn immediately after birth), pregnant women, and their partners in the Madrid Autonomous region [13]. In another study performed on pregnant women living in Eastern China, maternal blood and urine samples were obtained during the third trimester of pregnancy [14]. Parenteral transfer of Cd, Hg, and Pb by detecting them in maternal and cord blood samples (plasma and red blood cells), as well as in child postnatal blood samples of some African American mother-infant pairs randomly selected from the Boston Birth Cohort, was studied [15]. These works provide support for tobacco smoke and fish consumption as important preventable sources of heavy metal exposure in newborns.

### 1.2. Effects on Organs and Systems

Following toxic-metal exposure, many body organs and/or systems can be damaged [16]. The primary target organs of elemental Hg are the brain and the kidneys [17]. Childhood Cd exposure seems to adversely affect kidney function in rural Bangladesh [18]. Exposure to As, Cd, Pb, and Hg has been shown to produce some deleterious effects on the hematological system [19]. Pb and Cd affect blood, liver, and kidneys through the induction of oxidative stress [6]. In addition, since the brain is an organ that is especially vulnerable to the effects of ROS because of its oxygen demand and its abundance of perioxidation-susceptible lipid cells, the role of ROS in the pathophysiology of neurodegenerative diseases (ND) seems to be central [20].

### 1.3. Effects on Cells

Many dangerous effects of toxic metals have been reported at human cellular level. Indeed, Pb is known to alter the membrane composition of phospholipids in human erythrocytes, to inhibit transmethylation, and to exasperate phospholipid peroxidative damage [21]. Pb-induced neurodegeneration has been tested in neuronal PC12 cells that developed oxidative stress and apoptosis, aggravated by ethanol coexposure [22]. Pb damage on PC12 cells was propagated to neighboring cells through ROS-mitochondria-dependent apoptotic signaling via gap-junctional intercellular communication [23]. Heavy metal-induced alterations on kidney intercellular junctions have been also reported [24].

Considering the serious damage to human health caused by toxic-metal exposure, we should examine how this contamination can be determined in humans.

## 2. Assessment of Toxic-Metal Presence in Humans

Inductively Coupled Plasma Mass Spectrometry (ICP-MS) is the method currently used to evaluate toxic-metal levels in blood and/or urine samples in humans. The same method has been efficiently used to evaluate metal concentrations in hair samples [25].

After “acute” intoxication, determination of toxic metals in blood and urine samples represents a useful tool to assess the presence of such metals in humans. Strong correlation has been found between Cd in kidney and Cd in urine and blood in an environmental exposed population [26]. As reported for Hg species, rats exposed to thimerosal or methylmercury by gavage displayed high blood levels of total Hg, which progressively reduced from 6 to 120 hours after exposure. Tissues collected the fifth day after exposure revealed different forms of Hg (inorganic, methylmercury, or ethylmercury) in brain, liver, heart, and kidney [27]. However, the increase of blood toxic-metal values reflects only recent acute exposure, as seen for Pb [28]. Toxic metals rapidly move from the blood to many tissues where they are distributed, such as in the central nervous system (CNS). Accumulated metals can remain therein for many years, adversely affecting cell and tissue function. To remove sequestered toxic metals from the blood (where toxic metals accumulate following acute exposure) or human organs (where the same metals accumulate following chronic exposure) the only way is to bind such metals to chelating agents, with the aim of forming complexes. The routes of administration are oral, intramuscular (IM), and intravenous (IV). The complexes can be excreted especially by the kidneys. Indeed, toxic-metal levels can be examined in urine samples collected from patients, following the “challenge” with an opportune chelating agent.

Once the presence of toxic metals due to acute or chronic exposure has been determined, a second question arises: how can we remove these contaminating toxic metals? The most effective ways seem once again to be oral, IM, or IV administration of chelating agents to favor the elimination of toxic metals in the urine or in the faeces through the bile route. Chelation therapy plays a central role in modern medicine and pharmacology. Continuous studies with laboratory animals and extensive clinical experience show that acute or chronic intoxication with a variety of metals can be considerably improved by the administration of a suitable chelating agent [29]. As EDTA is able to bind Fe^+++^ but not Fe^++^ [30], in this review we have considered chelators able to remove iron excess, due to the clinical consequences of its overload, also in consideration of the role of Fe in neurodegeneration.

## 3. Chelating Agents

### 3.1. Iron (Fe) Excess in Thalassemia and ND

Acute Fe intoxication has been treated successfully with chelation therapy using deferoxamine. A woman who ingested ferrous sulphate in a suicide attempt was treated with the specific iron chelator deferoxamine, which binds ferric iron forming a water-soluble compound that is rapidly excreted by the kidney causing urine discoloration [31]. The important problems related to chronic Fe-overload observed in thalassemia patients can be overcome using chelating agents such as deferiprone, deferasirox, and deferoxamine [32]. Recently, combined treatment with oral deferiprone and subcutaneous desferoxamine twice weekly was shown to be a safe and effective alternative to chelation monotherapy in transfusion-dependent beta-thalassemia children [33]. The use of combined Fe chelators to prevent Fe-overload cardiomyopathy in thalassemia has been reported [34, 35]. Moreover, deferiprone is considered similar to deferoxamine in the treatment of thalassemia intermedia [36]. In addition, it has been shown that the oral Fe chelator deferasirox is able to inhibit NF- (nuclear factor-) kB dependent transcription without affecting its proximal activation, resulting in reduced TNF production from T cells stimulated in vitro. These results suggest that the hematopoietic improvement observed in deferasirox-treated patients affected by myelodysplasia and aplastic anemia might reflect an anti-inflammatory effect mediated through inhibition of the transcription factor NF-kB and support the therapeutic targeting of this pathway [37].

Much evidence has now been accumulated regarding the involvement of Fe in various ND as well as potential therapy with Fe chelators to retard their progression [38]. With ageing, there is an elevation of brain Fe (within ferritin and neuromelanin) with no apparent adverse effects in the frontal cortex, caudate nucleus, putamen, substantia nigra, and globus pallidus. However, excessive amounts of Fe in these specific regions, or in specific intracellular compartments of the brain, lead to ND. Indeed, redox-active metal ions, such as Fe and copper (Cu), can generate oxidative stress through production of ROS and nitrogen species. The one-electron reduction of H_2_O_2_ by Fe^2+^ (the well- known Fenton reaction) produces Fe^3+^ and the hydroxyl radical OH^∙^, which can react with a wide number of cellular constituents. This results in protein misfolding, amyloid production, and the formation of insoluble protein aggregates contained within inclusion bodies. Dysregulation of Fe metabolism, associated with cellular damage and oxidative stress, is reported as a common event in Alzheimer's disease (AD) and Parkinson's disease (PD) [39]. This will activate microglia, leading to neuroinflammation, which plays an important role in the progression of ND as activated microglia release proinflammatory cytokines that can damage neuronal cells. The evidence for metal involvement in PD and AD, as well as Friedreich's ataxia and multiple sclerosis (MS), suggests study of the effects of Fe chelators in patients affected by these diseases [38]. Brain Fe homeostasis is increasingly being recognized as a potential target for the development of drug therapy in age-related disorders. Deferiprone slightly improved motor symptoms in PD patients after 12 months of treatment, while the Fe content in the substantia nigra significantly decreased on magnetic resonance imaging (MRI) evaluation [40].

Excessive Fe can be toxic, and its accumulation in MS patients is generally thought to be detrimental. However, Fe maintains oligodendrocyte and myelin integrity and facilitates their regeneration following injury. The extracellular matrix, a key regulator of remyelination, might also regulate Fe levels. Histological and MRI studies have investigated changes in Fe distribution associated with MS. Treatment targeting Fe in MS patients must balance the trophic and toxic properties of Fe, providing sufficient Fe levels for remyelination and repair, while avoiding excesses that might overwhelm homeostatic mechanisms and contribute to damage [41]. In MS patients, the role of Fe in neurodegeneration has been highlighted, suggesting the use of Fe chelators together with neuroinflammation inhibitors [41].

### 3.2. Toxic-Metal Excess

The toxic metals that most frequently induce burdens in humans are Pb, Al, Hg, Cd, As, and Ni.

Other less-common toxic metals are antimony (Sb), barium (Ba), beryllium (Be), bismuth (Bi), cesium (Cs), gadolinium (Gd), palladium (Pd), platinum (Pt), tellurium (Te), thallium (Tl), thorium (Th), tin (Sn), tungsten (W), and uranium (U); Cr is a carcinogenic heavy metal responsible for DNA damage and mutations [42].  Table 1 reports (for each toxic metal) the target organs and/or apparatus, the sources of toxic metals (which are always represented by ingestion, inhalation, or dermal absorption; of note, gadolinium, when used as contrast agent, and platinum, when used as chemotherapeutic agent, could be IV administered), and the toxic doses of each metal.  Table 2 reports the most important intoxication symptoms and diseases.

### 3.3. Chelating Agents

Many agents were used in the past to remove metal intoxications, such as penicillamine, British anti-Lewisite (BAL or Dimercaprol), 2,3-dimercapto-1-propane sulfonic acid (Unithiol, DMPS), and meso-2,3-dimercaptosuccinic acid (Succimer, DMSA), other than EDTA. D-Penicillamine has been used to remove Hg, Pb, and Cd other than Cu in excess [43] and in association with DMPS in Wilson's disease [44]. BAL has been used in the management of heavy metal poisoning. In poisoning cases with elemental and inorganic Hg salts, BAL may be administered IM. DMSA and DMPS are administered orally or with IV injection and are currently used as chelating agents in Hg poisoning [45].

The human burden of all the reported toxic metals can be highlighted with the chelating agent EDTA using the so-called “chelation test.” As an acid, EDTA (ethylenediaminetetraacetic acid) has been used in clinical ophthalmology to treat band-shaped keratopathy [46]. In current clinical practice, it exists as sodium edetate (Na_2_EDTA) and as calcium disodium edetate (CaNa_2_EDTA), which is frequently reported as CaEDTA. The “challenge” with EDTA or the “chelation test” highlights the presence of toxic metals in urine samples recovered from patients. Patients are invited to collect urine samples before and after intravenous treatment with the chelating agent CaNa_2_EDTA (2 g/10 mL diluted in 500 mL physiological saline), referred to in the present paper as EDTA. EDTA is slowly administered intravenously (infusion lasts about 2 hours) and the time of urine collection following chelation lasts 12 h. Toxic-metal urine contents are expressed in micrograms (*μ*g) per g creatinine [47]. Details regarding “chelation test” have already been reported [48].

### 3.4. Usefulness of EDTA as Chelating Agent in Clinical Practice

The following cases reported in the literature confirm the usefulness of EDTA in clinical practice. In 1997, severe lead poisoning in humans was reported in rural Albania. Twenty-three people exhibiting signs of lead intoxications recovered following intensive CaEDTA chelation therapy [49]. EDTA chelation therapy was used by French researchers, in association with USA researchers, to remove contaminating metals and to decrease free-radical production in humans, as reported in a study published in 2005 [50]. The authors showed that the addition of high-dose (5 g) vitamin C to EDTA chelation solutions induced acute prooxidant effect, as monitored by lipid, protein, and DNA oxidative markers, but that long treatment (e.g., multiple sessions of EDTA chelation therapy) protected against oxidative damage, despite the presence of vitamin C. Moreover, in 2009 the French group showed that EDTA chelation therapy, without added vitamin C, decreased oxidative DNA damage and lipid peroxidation [51]. CaEDTA was used to avoid damage from acute Pb poisoning in an infant who received traditional Omani medicine (which was found to contain high Pb) for constipation [52]. Successful treatment with EDTA of extreme acute lead intoxication in a child was carried out in the Slovak Republic [53]. More recently, in Italy chelation with EDTA was successfully used to treat four patients exposed to occupational poisoning in two Chinese battery recycling plants [54]. The use of CaEDTA, rather than Na_2_EDTA, seems to be safer in the treatment of lead-intoxicated children [55]. CaEDTA chelation therapy has been used to remove Cd intoxication in association with reduced glutathione (GSH) [56]. GSH plays a key role in cell resistance against oxidative damage by providing enzymes involved in ROS metabolism with reducing equivalents, by eliminating potentially toxic oxidation products, and by reducing oxidized protein thiols [57]. Recently, we showed that CaNa_2_EDTA treatment of patients affected by Al burden significantly reduced Al intoxication [48]. Moreover, the efficacy of long-term chelation therapy has been shown in the removal of chronic Al intoxication [58].  Table 3 reports the more important clinical features of chelating agents. EDTA at the dose of 2 g/week with slow IV infusion (over around 2 hours) did not produce side effects in humans. In the past, when Na_2_EDTA was used at the dose of 6 g/week, an increase in parathyroid hormone and a decrease in serum bone alkaline phosphatase were seen [59]. At the dose of 2 g once a week, in my experience EDTA has no side effects.

## 4. Usefulness of EDTA Chelation Therapy in Cardiovascular Diseases (CVD) and Neurodegenerative Diseases (ND)

### 4.1. CVD

Na_2_EDTA is considered more able to chelate Ca^++^ than CaNa_2_EDTA and has been used to treat atherosclerosis, chronic inflammation related to endothelial dysfunction, as EDTA is thought to operate by scavenging calcium from fatty plaques. Indeed, EDTA chelation therapy using Na_2_EDTA has been used in the past and is still used today, to treat patients with coronary disease [60].

A recent trial assessed that in stable postmyocardial infarction patients the combination of oral high-dose vitamins and EDTA chelation therapy, compared with a double placebo, reduced clinically important cardiovascular events to an extent that was both statistically significant and of potential clinical relevance [61]. Chelation infusion contents contained a variable dose of ethylenediaminetetraacetic acid up to a maximum of 3 g depending on estimated glomerular filtration rate and were Na_2_EDTA based. A regimen of 40 infusions was carried out [62]. The results of this clinical trial (which showed that a metal chelator reduced cardiovascular events) highlight the potential connection between metal pollutants and CVD. Indeed, Hg, Pb, Cd, and As have been shown to display epidemiologic and mechanistic links to atherosclerosis and CVD, suggesting that environmental metal pollution might be a potent and modifiable risk factor for atherosclerotic disease [63]. Epidemiological evidence suggests that Cd and As exposure are associated with CVD incidence and mortality [63, 64]. Diabetes mellitus is a well-known risk factor for early CVD. EDTA chelation affects both transition and toxic metals. In fact, transition metals like Cu and Fe play important roles in the oxidative stress pathway, which is involved in insulin resistance, whereas the metals Pb and Cd are particularly toxic for the cardiovascular system. Recent findings therefore suggest the use of EDTA chelation therapy in the treatment of CVD, especially in diabetic patients [65].

### 4.2. ND

The role of prenatal and postnatal exposure to environmental factors can lead to the onset of ND in later life. Neurotoxic metals such as Pb, Hg, Al, Cd, and As, as well as certain pesticides and metal-based nanoparticles, have been involved in AD, due to their ability to increase beta-amyloid peptide and the phosphorylation of Tau protein, causing the senile amyloid plaques and neurofibrillary tangles that are characteristic of AD. Exposure to Pb, manganese (Mn), solvents, and pesticides has been related to certain PD hallmarks, such as mitochondrial dysfunction, alterations in metal homeostasis, and the aggregation of alpha-synuclein proteins, a key constituent of Lewy bodies that are a crucial factor in PD pathogenesis [66]. Chronic methyl-Hg exposure causes symptoms similar to those observed in amyotrophic lateral sclerosis (ALS) and AD. Indeed, Hg and its salts are able to induce depletion of GSH, mitochondria breakage, and increased lipid peroxidation of protein and DNA in the brain [67]. EDTA chelation therapy, whose usefulness has been shown in the elimination of Al intoxication [48], can therefore be recommended for the removal of other toxic metals responsible for ND development. The beneficial effect of EDTA treatment has been demonstrated by the neurological relief of symptoms such as spasticity, fatigue, and relapse in patients intoxicated by Al and affected by ND [48, 58, 68]. EDTA has been shown to be useful in attenuating peripheral blood cell damage in workers exposed to Pb [69]. The therapeutic effect of EDTA in experimental models of MS has been demonstrated, where EDTA was able to reduce demyelination plaques in intraperitoneally treated mice possibly in relation to the antioxidant and anti-inflammatory effects of EDTA [70].

## 5. Kidney Protection in EDTA Treatment

Cases of acute tubular necrosis have been reported following the early clinical use of EDTA involving very large doses [71]. At the doses now used clinically, EDTA effectively delays the progression of chronic kidney disease in patients with measurable body Pb burdens, as reflected by increased levels of estimated glomerular filtration rate and creatinine clearance rate [71].

EDTA treatment has been shown to slow down the progression of experimental Pb nephropathy [72]. Indeed, renal alteration produced in male Wistar rats during chronic Pb poisoning showed hypertrophy and vacuolization of medium and small arteries; mucoid edema and muscular hypertrophy in arterioles; loss of cell brush borders, cell loss, and intracellular inclusion bodies in the proximal tubules; fibrosis and the presence of infiltrates in the interstitial components. In rats treated intraperitoneally for five days with CaNa_2_EDTA such alterations were delayed. We previously demonstrated the efficacy of EDTA treatment in improving renal function in an experimental model of renal ischemia/reperfusion (I/R) [73]. Kidney I/R is an endothelial injury similar to what occurs in coronary disease. Moreover, such kidney protection has been shown in humans. Many papers positively describe clinically prolonged EDTA treatment in patients affected by chronic renal diseases (affected or not by diabetes), following or not Pb intoxication [74–78]. More specifically, researchers have shown that (i) low-level environmental Pb exposure may accelerate progressive renal insufficiency in patients without diabetes suffering from chronic renal disease; (ii) low-level environmental Pb exposure accelerates progressive diabetic nephropathy, and Pb-chelation therapy can decrease its rate of progression in patients with type-II diabetes; (iii) repeated chelation therapy might improve renal function and slow the progression of renal insufficiency in nondiabetic patients. Other toxic metals, such as Cd, Cr, Hg, and U, have previously been associated with nephropathy [79]. Noteworthily, all these metals are chelated by EDTA.

## 6. Passage of EDTA across the Blood-Brain Barrier

Some authors maintain that EDTA cannot cross the blood-brain barrier. Passage across the blood-brain barrier is physiologically possible into the circumventricular organs. However, new transport concepts for molecules and cells (inflammatory and neoplastic) under inflammatory conditions have been reported [80]. More recently, clearance of amyloid-beta peptide across the blood-brain barrier, the ability of an encephalic arbovirus to cross the same barrier, and barrier disruption by the streptococcus responsible for neonatal meningitis have been described [81–83]. If viruses, bacteria, and metastatic and inflammatory cells can cross the blood-brain barrier why can EDTA not cross it? Neuroinflammation in patients with ND relates to cytokine-dependent increase of blood-brain barrier permeability. Such a condition is possibly present also in healthy patients following pathogenic infection. The amelioration of neurologic symptoms in patients affected by ND treated with Fe chelators suggests they can cross the blood-brain barrier and remove iron deposition in the brain [38]. EDTA has a molecular weight lower than that of deferoxamine, an important iron chelator for thalassemia patients, and it is feasible that EDTA also can cross the blood-brain barrier especially in patients with neuroinflammation. In a recent paper, Louveau et al. describe the discovery of the CNS lymphatic system (functional lymphatic vessels lining the dural sinuses). These structures express all of the molecular hallmarks of lymphatic endothelial cells, are able to carry both fluid and immune cells from the cerebrospinal fluid, and are connected to the deep cervical lymph nodes. This discovery sheds new light on the etiology of neuroinflammatory diseases and ND [84] and possible new therapeutic strategies.

## 7. Usefulness of EDTA to Chelate Toxic Metals

**Figure 1:**
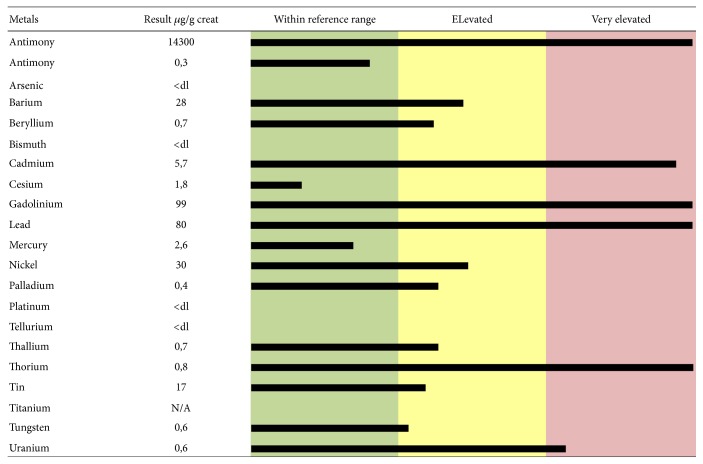
Toxic-metal levels measured in the patient's urine collected during the 12 hours following EDTA “challenge” (chelation test) reported in micrograms (*μ*g) per g creatinine. The 48-year-old female patient was affected by multiple sclerosis.

**Figure 2:**
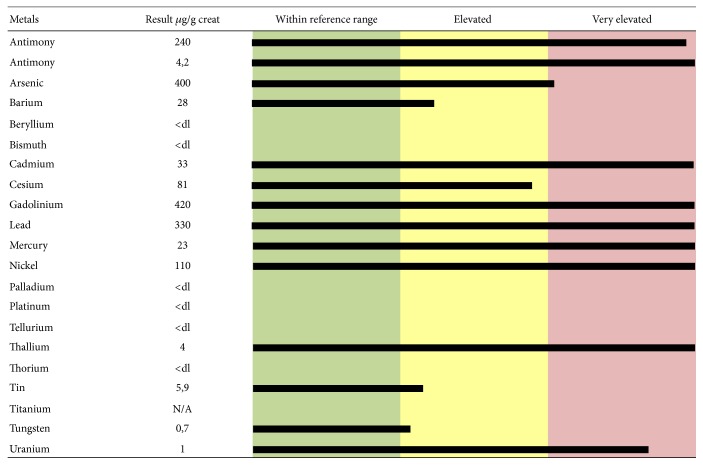
Toxic-metal levels measured in the patient's urine collected during the 12 hours following EDTA “challenge” (chelation test) reported in micrograms (*μ*g) per g creatinine. The 61-year-old male patient was affected by idiopathic progressive spastic paraparesis.

Many papers report that EDTA is able to chelate Pb, Cd, zinc (Zn), and Ca. The EDTA-metal chelate is water soluble and is typically excreted in the urine. It can form strong covalent bonds with the metals and increase urinary excretion. EDTA can chelate all toxic metals, as shown in two urine mineralograms reported as examples (Figures 1 and 2).  Figure 1 shows toxic-metal values in urine samples taken after chelation test and expressed as *μ*g per g creatinine, from a 48-year-old female patient affected by MS. Elevated levels of Ba, Be, Ni, Pd, Tl, Sn, and W and extremely elevated levels of Al, Cd, Gd, Pb, Th, and U are highlighted.  Figure 2 shows toxic-metal values in urine samples taken from a 61-year-old male patient affected by idiopathic progressive spastic paraparesis. Elevated levels of Ba, Cs, Sn, and W and extremely elevated levels of Al, Sb, As, Cd, Gd, Pb, Hg, Ni, Th, and U are highlighted. Before EDTA challenge, the urine samples did not reveal the presence of toxic metals (data not shown). The elevated intoxication (in terms of both highest levels of toxic metals and high number of toxic metals involved) shown by these two patients correlates with their serious clinical manifestations (extreme walking impairment, gait ataxia, leg and arm spasticity, paresthesia, disturbance of fine motor skills, etc.). The results displayed by more than 1,000 urine mineralograms show a constant relationship between highest toxic-metal burden and most serious symptoms of ND that were successfully improved by EDTA chelation therapy (personal unpublished data).

## 8. Concomitant Treatment with Antioxidants

Chelation therapy can be efficiently associated with daily antioxidant treatment to improve the endogenous detoxification ability of each patient. The use of antioxidant treatment can improve endogenous mechanisms endowed with antioxidant functions, especially in ND patients. The antioxidant treatments could be glutathione (Ultrathione, 500 mg/day), alone or together with multivitamin complexes, amino acid and mineral mixtures, or probiotics. We have already shown how chelation therapy with CaNa_2_EDTA, associated with the antioxidant compound Cellfood, can significantly improve the blood parameters of patients affected by ND, as well as those of unaffected healthy patients (controls) [68]. Indeed, after three-month chelation+Cellfood administration, oxidized LDL (oxLDL) decreased, ROS levels were significantly lower, and total antioxidant capacity (TAC) and glutathione levels were significantly higher than following chelation+other above reported antioxidant treatments. Moreover, homocysteine metabolism also improved in both groups (Cellfood and other antioxidants), also lasting three months both in ND patients and in controls. Cellfood (Eurodream, La Spezia, Italy) is an antioxidant nutritional supplement containing 78 ionic/colloidal trace elements and minerals combined with 34 enzymes and 17 amino acids, all suspended in a solution of deuterium sulphate that is efficient in protecting against damage in vitro [85]. In a previous study, it was shown that Cellfood treatment in vitro increased mitochondrial metabolism in endothelial cells [86]. New interest is emerging in the role of mitochondria in disease development and progression and also as a target for environmental toxicants. Respiratory dysregulation has been linked to cell death and helps increase the onset of neurodegenerative diseases, such as PD and AD. The mechanisms underlying the sensitivity of the mitochondrial respiratory complexes to redox modulation, as well as the effects of environmental contaminants that have mitochondrial toxicity, have recently been examined and discussed [87]. Protection of mitochondria against damage due to specific xenobiotics can be a useful tool to help avoid disease. The lack of significant studies on the efficacy of associated treatment using chelating agents and antioxidants to improve oxidative stress parameters in humans highlights the reported results.

## 9. Concluding Remarks

Recent research suggests that EDTA chelation may be a well-tolerated and effective treatment method for postmyocardial infarction patients [88]. Toxic metals are pollutants that can represent cardiovascular risk factors and are involved in the development of vascular complications, especially in diabetic patients [89]. Indeed, EDTA chelation therapy can be promoted as treatment for heart disease, particularly in patients with diabetes [90]. Toxic metals also exert an important role in the pathogenesis of ND. EDTA chelation therapy has been shown to improve neurologic symptoms in ND patients [48, 68], and results are further enhanced by the use of antioxidants. EDTA chelation therapy, at the dose of 2 g/week slowly injected IV in adults, does not provoke side effects, can be used for a long period to ameliorate both acute and chronic intoxications, and is able to remove all toxic metals and reduce oxidative stress injury and inflammation in blood vessel walls [50, 51, 60, 65, 73, 90]. EDTA treatment represents an important option in the treatment of ND and CVD associated with metal burden. No interaction is known regarding the drugs commonly used for CVD or ND with EDTA.

## Figures and Tables

**Table 1:** Target organs/apparatus, sources, and toxic levels for each toxic metal are reported.

	Main target organs or apparatus									Main assimilation way	Toxic levels *µ*g/g creatinine
	Kidney	SNC	Liver	Gastrointestinal system	Respiratory system	Skin	Cardiovascular/hematopoietic system	Oral/nasal mucosa	Musculoskeletal system	Ingestion (I) Inhalation (IH) Dermal absorption (DA)	
Aluminum		x			x				x	I, IH	35,00
Antimony		x		x	x	x	x		x	I, IH, DA	0,30
Arsenic		x	x	x	x	x	x			I, IH	108,00
Barium		x		x	x		x		x	I, IH	7,00
Beryllium	x		x		x	x				I, IH	1,00
Bismuth	x	x	x		x	x		x	x	I, IH, DA	10,00
Cadmium	x	x	x	x	x		x		x	I, IH	0,80
Cesium	x	x	x	x	x	x	x			I, IH, DA	9,00
Gadolinium				x	x				x	I	0,30
Lead	x	x		x		x	x		x	I, IH, DA	2,00
Mercury	x	x	x	x	x		x	x		I, IH, DA	3,00
Nickel		x			x	x		x	x	I, IH, DA	10,00
Palladium					x	x				I, DA	0,30
Platinum	x			x		x				IH, DA	1,00
Tellurium				x	x	x		x		I, IH	0,80
Thallium		x		x		x	x			I, IH	0,50
Thorium		x	x		x					I, IH	0,03
Tin		x		x		x				I, IH, DA	9,00
Titanium		x		x	x	x		x		I, IH, DA	15,00
Tungsten			x		x	x				I, IH	0,40
Uranium	x	x	x		x	x			x	I, IH, DA	0,03

**Table 2:** The more common symptoms and/or diseases developed following intoxication are reported for each toxic metal. These are a free postediting from Zhou et al. [16].

	Symptoms and diseases
Aluminum	Fatigue, hypophosphatemia, increased prothrombin time, and porphyria.
Antimony	Nausea, anorexia, metallic taste, fatigue, and muscle weakness. Hypotension, cardiac pain (like angina pectoris), and faulty ventricular polarization. Irritation of respiratory tissues, pneumoconiosis. “Antimony spots” on the skin.
Arsenic	Fatigue, malaise. Eczema or allergic-like dermatitis. Skin hypopigmentation, white striae on fingernails, hair loss, and stomatitis. Increased salivation and garlic-like breath. Peripheral neuropathy. Myocardial damage, hemolysis, and aplastic anemia with leukopenia.
Barium	Tingling in the extremities, abnormal reflexes, and paralysis. Vomiting, diarrhea. Arrhythmia, hypertension. Respiratory arrest/failure.
Beryllium	Hypoproteinemia, anemia. Liver and kidney damage. Chest pain, cough, and fatigue. Granulomatous, lung disease, lung cancer.
Bismuth	Constipation or bowel irregularity, colitis. Foul breath. Skin pigmentation changes and gum pigmentation (blue-black), gingivitis, and stomatitis. Erythema and skin sores, irritation of mucous membranes. Nephritis, nephrosis, and hepatitis. “Bismuth encephalophy” with mental confusion, clumsiness, myoclonic jerks, tremors, and dysarthria (osteoarthropathy).
Cadmium	Hypertension. Microcytic-hypochromic anemia (not responsive to iron supplementation). Proteinuria with abnormally high excretion of beta-2 microglobulin.
Cesium	Ventricular arrhythmias, cardiotoxicity. Headache, nausea, and epileptic seizures.
Gadolinium	Abdominal cramps, diarrhea. Lethargy, muscular spasms, and respiratory collapse. Irritation of the skin and eyes.
Lead	Loss of appetite, constipation. Poor hemoglobin synthesis, anemia. Nephrotoxic effects with impaired renal excretion of uric acid. Tremors, neuropathy, and encephalopathy.
Mercury	Decreased senses of touch, hearing, vision, and taste, metallic taste in mouth. Fatigue or lack of physical endurance. Increased salivation. Anorexia, numbness and paresthesias, headaches, irritability, and excitability. Tremors and incoordination, psychoses, and manic behaviors. Anemia. Hypertension.
Nickel	Headache, vertigo. Muscle weakness. Nausea, vomiting. Palpitations. Itchy skin and dermatitis, eczema. Panallergy with asthma, conjunctivitis, rhinitis, and sinusitis.
Palladium	Skin, eye, and respiratory irritation and allergic contact dermatitis.
Platinum	Nausea, vomiting. Dyspnea and wheezing. Dermatitis, development of chronic allergic reactions (“platinosis”). Nephrosis. Anemia, thrombocytopenia, and leukopenia.
Tellurium	Eye and respiratory irritation. Drowsiness, headache. Dry mouth, metallic taste, garlic-like odor of breath, sweat, and urine. Abdominal pain, nausea, vomiting, and anorexia. Respiratory depression. Circulatory collapse. Bluish/black patches on the skin.
Thallium	Nausea, gastrointestinal distress, diarrhea, and weight loss. Proteinuria. Fatigue. Mental confusion, memory loss, psychoses, and peripheral neurological signs: paresthesias, myalgias, tremor, and ataxia. Hair loss with sparing of pubic and body hair and a lateral fraction of eyebrows, alopecia.
Thorium	Respiratory distress and pneumonia, pulmonary hypertension, and fibrosis.
Tin	Reduced sense of smell, headache, fatigue, ataxia, and muscle weakness. Irritation of contacted tissues (eyes, skin, bronchial tubes, or gastrointestinal tract).
Titanium	Chest tightness and respiratory difficulty. Eye skin irritation. Neuroinflammation by titanium dioxide nanoparticles. Hypersensitivity reaction.
Tungsten	Lung diseases (pneumoconiosis, cancer). Eczema, pruritus, folliculitis, and neurodermatitis.
Uranium	Fatigue. Hair loss, alopecia, and dermatitis. Increased locomotor activity. Perturbation of the sleep-wake cycle, decreased memory, and increased anxiety.

**Table 3:** Characteristics of chelating agents used in clinical practice.

	Route of administration	Adult dose	Route of metal complex excretion	Side effects
BAL	IM	5 mg/kg/day	Urine, bile, faeces, lungs	Nausea, vomiting, hypertension, tachycardia, headache

Deferiprone	Oral	50–100 mg/kg/day	Urine	CNS toxicity, lenticular opacities, arthropathy

Deferoxamine	IV	50 mg/kg/day	Urine	Nausea, weight loss, ocular toxicity
	Subcutaneously	20 mg/kg/day		

Deferasirox	Oral	35 mg/kg/day	Faeces, urine	Abdominal pain, nausea, vomiting, elevation of liver enzymes

DMPS	Oral	300 mg three times a day	Urine	Rash, nausea, leukopenia
	IV	until 1500 mg/day		
	IM	20 mg/kg/day		

DMSA	Oral	30 mg/kg/day	Urine, bile, faeces, lungs	Gastrointestinal disorders (GI), skin rashes, flu-like symptoms
	IV	10 mg/kg three times a day		

EDTA	IV	2 gr/week	Urine	None^*∗*^

D-Penicillamine	Oral	5–20 mg/kg/day	Urine	Degenerative dermopathy, thromboleukocytopenia, GI

^*∗*^When used once a week at the dose reported and intravenously injected in about 2 hours.

DMPS: 2,3-dimercapto-1-propane sulphonic acid.

DMSA: meso-2,3-dimercaptosuccinic acid.

EDTA: CaNa_2_ ethylenediaminetetraacetic acid.
